# Assessment of the Diagnostic Utility of GeneXpert Mycobacterium tuberculosis/Rifampicin (MTB/RIF) Assay in the Suspected Cases of Tuberculous Meningitis

**DOI:** 10.7759/cureus.37761

**Published:** 2023-04-18

**Authors:** Sakshi Patel, Malti Dadheech, Anand K Maurya, Jitendra Singh, Shashank Purwar, Nirendra Rai, Radha Sarawagi, Ankur Joshi, Sagar Khadanga

**Affiliations:** 1 Department of Microbiology, All India Institute of Medical Sciences, Bhopal, Bhopal, IND; 2 Department of Translational Medicine Center, All India Institute of Medical Sciences, Bhopal, Bhopal, IND; 3 Department of Neurology, All India Institute of Medical Sciences, Bhopal, Bhopal, IND; 4 Department of Radiodiagnosis, All India Institute of Medical Sciences, Bhopal, Bhopal, IND; 5 Department of Community and Family Medicine, All India Institute of Medical Sciences, Bhopal, Bhopal, IND; 6 Department of General Medicine, All India Institute of Medical Sciences, Bhopal, Bhopal, IND

**Keywords:** cerebrospinal fluid, mycobacterium tuberculosis, xpert mtb/rif, extrapulmonary tuberculosis, tuberculous meningitis

## Abstract

Introduction: Tuberculous meningitis (TBM) is a manifestation of extrapulmonary tuberculosis (EPTB) caused by *Mycobacterium tuberculosis *(MTB). The central nervous system is involved in about 1%-2% of all current tuberculosis (TB) cases and about 7%-8% of all EPTB. if not treated early, TBM leads to a high rate of neurological sequelae and mortality.

Objective: This study aimed to assess the diagnostic performance of the GeneXpert MTB/rifampicin (RIF) assay in patients with TBM.

Methods: A total of 100 suspected TBM cases were enrolled from various departments at tertiary care hospital, Bhopal, Madhya Pradesh, India, and classified as definite, possible, or probable TBM. The clinical samples were tested for microbiological and other cerebrospinal fluid (CSF) testing.

Results: Out of 100 cases, 14 (14%) were classified as definite TBM, 15 (15%) were having probable TBM, and 71 (71%) were having possible TBM. Out of a total of 100 participants, all were negative for acid-fast bacilli (AFB) staining. Of the 100 cases, 11 (11%) were positive by mycobacterium growth indicator tube (MGIT) culture, of which only four (36.36%) were positive by GeneXpert MTB/RIF. GeneXpert MTB/RIF detected three (3%) cases that were negative by MGIT culture. Ten (90.9%) of the 11 MGIT-positive culture isolates were found to be RIF sensitive while one (9.1%) was found to be RIF resistant. Three cases tested positive/sensitive by the GeneXpert MTB/RIF but negative by MGIT culture. Six (85%) of the seven GeneXpert MTB/RIF positive cases were RIF sensitive while one (15%) was RIF resistant. The sensitivity, specificity, positive predictive value (PPV), negative predictive value (NPV), and diagnostic accuracy were 36.36% (95% Confidence Interval (CI) (10.93% to 69.21%)), 96.63% (95% CI (90.46% to 99.30%)), 57.14% (95% CI (25.50% to 83.85%)), 92.47% (95% CI (88.70% to 95.06%)) and 90% (95% CI (82.38% to 95.10%)) for GeneXpert MTB/RIF assay, compared with MGIT culture as the reference standard.

Conclusion: Our study found that the sensitivity is lower when compared to culture, so using GeneXpert MTB/RIF alone is not recommended. Overall performance of GeneXpert MTB/RIF assay is noteworthy. The GeneXpert MTB/RIF assay is a potentially accepted test for obtaining an earlier diagnosis, and if it tested positive, the treatment should begin immediately. However, culture must be performed in GeneXpert MTB/RIF negative cases.

## Introduction

Tuberculous meningitis (TBM) is a manifestation of extrapulmonary tuberculosis (EPTB) caused by *Mycobacterium tuberculosis *(MTB) [1]. The central nervous system is involved in about 1%-2% of all current tuberculosis (TB) cases and about 7%-8% of all EPTB cases documented in immunocompetent patients [2,3]. TBM leads to a high rate of neurological sequelae and mortality. TBM is uncommon in affluent nations, with only 100 to 150 cases reported annually in the United States, accounting for less than 3% of the estimated 4,100 cases of bacterial meningitis yearly [4].

The exact prevalence of TBM in India is unknown, but according to India's official guidelines for the treatment of EPTB, TBM may be present in 1% of all TB cases [5]. TBM is more difficult to diagnose than other types of bacterial meningitis because symptoms do not appear as quickly as they do with classic bacterial meningitis, and it is a paucibacillary infection that is difficult to detect in cerebrospinal fluid (CSF) [6].

Laboratory diagnostic procedures are primarily culture-dependent (using liquid and/or solid culture media). Culture takes several weeks to yield observable bacterial replication, and the sensitivity is low, around 40%-50% [7,8]. Although automated systems such as mycobacterium growth indicator tube (MGIT) 960 culture system by Becton Dickinson have shown a reduced average time to yield results (18 days versus 38 days), clinicians cannot afford to wait for culture results before treating patients, as death is a distinct possibility if empirical therapy is not initiated [9]. Conventional diagnostic techniques for multidrug-resistant TB (MDR-TB) typically involve a lengthy and arduous process that requires a series of steps to isolate the mycobacteria in clinical samples, identify the MTB complex, and then in vitro analysis to determine the strain’s sensitivity to anti-tuberculous drugs [10,11]. One of the most difficult issues clinicians confront is the timely and accurate diagnosis of TBM, which requires a combination of clinical criteria, radiographic, and laboratory investigations [12]. When MTB cannot be detected in the CSF, an unconfirmed diagnosis of TBM is frequently made based on a combination of clinical symptoms, radiological and CSF findings [13,14].

Changes in TBM diagnosis have occurred over the last few decades. For the rapid onset of TB, genotypic methods have come into play. Genotypic methods are faster and have higher sensitivity and specificity. The GeneXpert MTB/RIF assay (Cepheid, Sunnyvale, CA, United States) is an automated closed-cartridge system for detecting MTB and rifampicin (RIF) resistance in less than two hours [15]. It was approved by the World Health Organization (WHO) in 2013 for the diagnosis of EPTB in adults and children, including CSF in patients with suspected TBM [6]. As a result, TB experts emphasize the importance of re-evaluating GeneXpert's performance and improving and standardizing CSF sample processing to order to increase sensitivity and specificity [6]. This study was aimed to assess the diagnostic performance of the GeneXpert MTB/RIF assay in patients with suspected TBM.

## Materials and methods

This cross-sectional study was conducted at the Department of Microbiology, All India Institute of Medical Sciences (AIIMS), Bhopal, Madhya Pradesh, India, from November 2020 to April 2022. Ethical clearance was obtained from the Institutional Human Ethics Committee-Post Graduate Research (IHEC-PGR) of the AIIMS, Bhopal, Madhya Pradesh, India (Ref Number: 2020/PG/Jan/13, dated November 17, 2020). The sample size of this study was 100 which was determined as the number of samples required for detecting an estimated prevalence of TBM cases with a relative precision of 5% and 5% of α error and 95% of CI.

All the suspected TBM cases presented at AIIMS, Bhopal, Madhya Pradesh, India, were included in the study. Patients who were not willing for the consent were excluded from the study. A total of 100 TBM-suspected cases in the age range 0-80 years were enrolled from the pediatrics, general medicine, and neurology departments of AIIMS, Bhopal. All demographic, clinical, and radiological details have been filled in the request form for all the suspected cases of TBM. In this study, we used Lancet Consensus Case Definition Criteria as per Marais et al. to categorize the TBM cases into three categories [16]. (i) Definite TBM cases with confirmation of acid-fast bacilli (AFB) in the CSF by culture, microscopy, and commercially available nucleic acid amplification test. (ii) Probable TBM cases with a diagnostic score of 10 or more without magnetic resonance imaging (MRI) or computed tomography (CT) scan or 12 or more with MRI or CT scan. (iii) Possible TBM cases have a diagnostic score of between 6 and 9 if cerebral imaging was not performed or between 6 and 11 if cerebral imaging was performed.

CSF samples were collected during lumbar puncture and a volume of 2-6 mL CSF sample was collected into sterile plastic containers with leak-proof screw caps. CSF was centrifuged at 3,000-3,500g for 15-20 minutes, and re-suspended sediments were used for smear preparation, MGIT inoculation, and GeneXpert MTB/RIF assay. The biosafety precautions were taken while handling the samples.

Ziehl Neelsen (ZN) staining

Three drops of the specimen were put on the slide with a pipette. The smear was heat fixed and stained with ZN staining method. Stained smears were examined using a bright field microscope with a 100x oil immersion field. In positive smears, MTB appeared straight/slightly curved, long slender, beaded red-colored, uniformly stained AFB. For labeling the smear as negative, a minimum of 100x oil immersion fields were observed [6].

Liquid culture using MGIT 960 TB system

Firstly, 800 µL volume of the antibiotic mixture of Polymyxin-B, Amphotericin-B, Nalidixic acid, Trimethoprim, Azlocillin (PANTA), and growth supplement solution was added to each MGIT tube, followed by 500 µL of CSF sample. After scanning the MGIT tube barcode, all inoculated tubes were loaded into the automated MGIT-960 system in the slot assigned by the instrument. All the positive flagged tubes were taken for further processing and all the negative flagged tubes were removed after the completion of 42 days of incubation. ZN staining was performed on all the flagged positive tubes. All the smears positive for AFB were further confirmed by MPT64 antigen card test as per the manufacturer’s instructions.

Confirmation of MTB by rapid MPT64 antigen card test

A total of 100 µL of culture broth was added to the well of the Ag MPT64 TB rapid kit cassette. Inoculated immunochromatographic cassettes (ICT) were kept at room temperature for 20 minutes in the Class II biosafety cabinet before being examined for the presence of control and test bands. The band in the “C” region confirmed test validity. An additional band in the “T” region was interpreted as positive for MPT64 Ag. Only one band in the “C” region and no band in the “T” region were considered negative for MPT 64 antigen. No band in the “C” region was interpreted as an invalid test.

GeneXpert MTB/RIF assay

A volume of 2 mL of sample buffer reagent was added to 1 mL of CSF centrifuged sediment, mixed well, and incubated for 15 min at room temperature. Then 2 mL of the mixture was transferred to the sample chamber in the cartridge. The cartridge was loaded in the GeneXpert instrument, and the results were obtained after about two hours. For MTB, the results were either “MTB detected” or “MTB not detected.” RIF drug results were reported as susceptible, resistant, or indeterminate. The results were classified as high, medium, low, or very low based on the cycle threshold (Ct) value of the MTB target DNA.

Drug susceptibility testing (DST) for RIF by MGIT

For MGIT-positive culture isolates, a drug susceptibility test for RIF was performed as per the manufacturer's instructions. Susceptibility testing was performed using a 1% proportional method and a drug concentration of 1.0 μg/mL [17]. The BACTEC MGIT 960 instrument automatically interpreted the readings and classified them as susceptible or resistant.

Other investigations

All patients underwent routine tests for TBM such as CSF cell count and radiological examination.

Data collection and statistical analysis

Throughout the study, all data were collected in the request case report form with a written consent form. The Excel sheet contained all of the information, including the demographic profile, clinical and radiological findings, and laboratory and CSF findings. The sensitivity, specificity, positive predictive value (PPV), negative predictive value (NPV), and diagnostic accuracy with a 95% confidence interval (CI) of the GeneXpert MTB/RIF assay were calculated in comparison to MGIT culture.

## Results

Over an 18-month period, 100 patients with suspected TBM presented to AIIMS, Bhopal (Madhya Pradesh), India. In correlation between Lancet Consensus Criteria for TBM and clinical parameters, 14 (14%) of the 100 cases were classified as definite TBM, 15 (15%) were having probable TBM and 71 (71%) were having possible TBM.

The average age of the definite TBM cases was 31.87 ± 20.22, probable cases were 21.97 ± 12.07 and that of the possible cases was 29.83 ± 22.08. Patients with definite and probable TBM were more likely to be female, whereas men were more likely to have possible TBM. A total of 31 patients were in the pediatric age group. Out of which two had definite TBM, 23 had possible TBM, and six had probable TBM. Fever was the most prominent clinical feature in 14 (100.0%) of the definite, 14 (93.3%) of the probable, and 71 (100.0%) of the possible TBM cases, followed by weight loss in 12 (85.7%) of the definite, 10 (66.7%) of the probable, 53 (74.6%) of the possible TBM cases and loss of appetite in 12 (85.7%) of the definite, 10 (66.7%) of the probable and 52 (73.2%) of the possible cases. As shown in Table 1, lethargy 44 (62.0%) and altered sensorium 48 (67.6%) were more likely to be present in definite TBM cases.

**Table 1:** Association between lancet consensus criteria for TBM and different clinical parameters

Parameters	Lancet Consensus Criteria for TBM		
	Possible Case (n = 71)	Probable Case (n = 15)	Definite Case (n = 14)
Age (Mean age)	29.83 ± 22.08	21.97 ± 12.07	31.87 ± 20.22
Gender			
Male	41 (57.7%)	5 (33.3%)	6 (42.9%)
Female	30 (42.3%)	10 (66.7%)	8 (57.1%)
Symptoms			
Fever	71 (100.0%)	14 (93.3%)	14 (100.0%)
Weight Loss	53 (74.6%)	10 (66.7%)	12 (85.7%)
Loss of Appetite	52 (73.2%)	10 (66.7%)	12 (85.7%)
Cough	20 (28.2%)	3 (20.0%)	5 (35.7%)
Dyspnea	9 (12.7%)	2 (13.3%)	2 (14.3%)
Meningeal Signs	9 (12.7%)	2 (13.3%)	5 (35.7%)
Cranial Nerve Palsies	9 (12.7%)	2 (13.3%)	2 (14.3%)
Papilledema	8 (11.3%)	6 (40.0%)	3 (21.4%)
Optic Atrophy	0 (0.0%)	1 (6.7%)	0 (0.0%)
Focal Neurological Deficits (other than Urinary/Faecal Incontinence)	22 (30.98%)	4 (26.7%)	3 (21.4%)
Focal Neurological Deficits-Urinary/Faecal Incontinence	15 (21.1%)	2 (13.3%)	2 (14.3%)
Altered Sensorium	48 (67.6%)	9 (60.0%)	11 (78.6%)
Lethargy/Drowsy/ Obtunded	44 (62.0%)	8 (53.3%)	11 (78.6%)
Abnormal Glasgow Coma Scale	19 (26.8%)	4 (26.7%)	8 (57.1%)
Headache	36 (50.7%)	8 (53.3%)	9 (64.3%)
Neck Stiffness	20 (28.2%)	4 (26.7%)	8 (57.1%)
Convulsions	24 (33.8%)	4 (26.7%)	4 (28.6%)
Chest Pain	4 (5.6%)	1 (6.7%)	1 (7.1%)
Previous History of TB	3 (4.2%)	0 (0.0%)	0 (0.0%)
Family History of TB	0 (0.0%)	0 (0.0%)	0 (0.0%)
HIV Positive	1 (1.40%)	0 (0.0%)	0 (0.0%)
Raised Intracranial Pressure	6 (8.45%)	3 (20.0%)	2 (14.2%)
Low Intracranial Pressure	1 (1.40%)	0 (0.0%)	0 (0.0%)
Chest X-ray/CECT chest			
Normal	38 (53.5%)	5 (33.3%)	3 (21.4%)
Abnormal (Consolidation, fibrosis, multiple miliary nodules, infiltrate, bronchiectasis and cavitatory changes)	5 (7.04%)	2 (13.3%)	5 (35.7%)
MRI			
Normal	5 (7.04%)	2 (13.3%)	0 (0.0%)
Abnormal (Hydrocephalus [Obstructive and non-obstructive], infarcts, Basal meningeal enhancement, basal hyperdensity, tuberculoma)	32 (40.07%)	7 (46.6%)	7 (50.0%)
CT Brain			
Normal	21 (29.5%)	3 (20%)	2 (14.2%)
Abnormal (Hydrocephalus [Obstructive and non-obstructive], infarcts, Basal meningeal enhancement, basal hyperdensity, tuberculoma)	17 (23.9%)	5 (33.3%)	6 (42.8%)
CSF: Appearance			
Thick Purulent	1 (1.4%)	0 (0.0%)	0 (0.0%)
Clear	69 (98.6%)	15 (100.0%)	14 (100.0%)
CSF: Cells			
Raised	22 (31.9%)	15 (100.0%)	12 (85.7%)
Normal	47 (66.1%)	0 (0.0%)	2 (14.3%)

The microbiological characteristics of 100 TBM suspected cases by ZN staining, MGIT culture, GeneXpert MTB/RIF and MGIT DST for RIF results summarized were in Table 2.

**Table 2:** Results of ZN microscopy, MGIT culture, GeneXpert MTB/RIF, and MGIT DST for RIF ZN: Ziehl Neelsen, MGIT: mycobacterium growth indicator tube, MTB/RIF: Mycobacterium tuberculosis/rifampicin, DST: drug susceptibility testing

Diagnostic tests		Specimens n=100 (%)
Smear Microscopy		
AFB Seen		0
No AFB Seen		100 (100%)
MGIT Culture		
Positive		11 (11%)
Negative		89 (89%)
GeneXpert MTB/RIF		
MTB Detected		7 (7%)
MTB Not Detected		93 (93%)
Rifampicin Sensitive		6 (85.72%)
Rifampicin Resistant		1 (14.28%)
Indeterminate		0
MGIT DST for Rifampicin		
Rifampicin sensitive		10 (90.90%)
Rifampicin Resistant		1 (9.10%)

The sensitivity, specificity, PPV, NPV and diagnostic accuracy for GeneXpert MTB/RIF assay was 36.36% (95% CI (10.93% to 69.21%)), 96.63% (95% CI (90.46% to 99.30%)), 57.14% (95% CI (25.50% to 83.85%)), 92.47% (95% CI (88.70% to 95.06%)), and 90% (95% CI (82.38% to 95.10%)) compared with MGIT culture as the reference standard. The results were summarized in Table 3.

**Table 3:** Diagnostic performance of GeneXpert MTB/RIF with MGIT culture as the reference standard

Statistics	Value	95% CI
Sensitivity	36.36%	10.93% to 69.21%
Specificity	96.63%	90.46% to 99.30%
Positive Predictive Value	57.14%	25.50% to 83.85%
Negative Predictive Value	92.47%	88.70% to 95.06%
Diagnostic Accuracy	90.00%	82.38% to 95.10%

## Discussion

TB is the world’s most dreadful infectious disease with high morbidity and mortality among people [18]. TBM among all forms of EPTB leads to the highest proportion of lethal sequelae [19]. Due to the paucibacillary nature of the disease, diagnosis of TBM is the greatest challenge [20]. Therefore, a rapid and accurate diagnostic modality is needed for the early detection of MTB.

According to the Lancet Consensus Criteria by Marais et al., 14 (14%) of the 100 recruited cases were definite, 15 (15%) were probable, and 71 (71%) were possible TBM cases in this study. In clinical research, Lancet Consensus Criteria comes up with strong and practical use of clinical case definition in 2010 [16].

All of the CSF samples in this study tested negative for direct ZN staining. The paucibacillary nature of the bacilli in the CSF samples could be one reason. Previous studies showed a low detection rate of 0%-20% for MTB in CSF specimens [21]. The possible reason could be that the intracellular bacilli cannot be easily stained by acid-fast dyes. Another reason could be that detecting mycobacteria in TBM cases necessitates a larger volume of CSF samples. Conventional ZN staining requires 10,000 bacilli per slide or per mL of specimen [22].

Culture methods are time-consuming and have a sensitivity of around 40%-50% [23]. Because liquid culture is more sensitive than solid culture, we used it as the gold standard for MTB detection [24]. Although automated systems like MGIT have shown a reduction in average time to yield results. As a result, the MGIT culture was used as a reference standard to compare the sensitivity and specificity of the GeneXpert MTB/RIF assay in the clinical specimen [25]. When compared to the reference standard (MGIT 960 automated culture system), the diagnostic sensitivity of the GeneXpert MTB/RIF assay was 36.36% (95% CI (10.93% to 69.21%)). Despite the fact that it is a rapid test, the diagnostic sensitivity of the GeneXpert MTB/RIF assay was found to be low in this study.

The possible reason for the low sensitivity of GeneXpert assay being a molecular test is low genetic material due to less number of bacilli in the sample [26]. Another reason could be the presence of inhibitors in the CSF sample. PCR inhibitors in CSF samples such as erythrocytes can cause errors in the results [27]. Alternatively, if the bacilli are not captured and lysed during the process, the results will be incorrect because the proficiency of the GeneXpert MTB/RIF assay is dependent on the capture of intact bacilli from the specimen within the cartridge [28].

The specificity of the GeneXpert MTB/RIF assay was 96.63% (95% CI (90.46% to 99.30%)) in this study. The PPV was 57.14% (95% CI (25.50% to 83.85%)), NPV was 92.47% (95% CI (88.70% to 95.06%)), and the diagnostic accuracy was 90% (95% CI (82.38% to 95.10%)). A study by Rufai et al., (2017) observed 55.1% (95%, CI: 40.2-69.3) sensitivity and 94.8% (95%, CI: 90.9-97.4) specificity of GeneXpert MTB/RIF assay in comparison to MGIT culture [26]. Vadvai et al. had observed sensitivity of 29% in CSF samples [29]. Metcalf et al. had observed sensitivity of 23% [6]. Three patients in this study were culture negative but GeneXpert MTB/RIF assay positive; the reason could be that the patients had a history of previous anti-tubercular treatment, and the dead bacilli DNA was detected by the GeneXpert MTB/RIF assay [26].

Seven cases were negative by GeneXpert MTB/RIF assay and direct ZN staining, but positive by MGIT 960 culture system. The very low bacillary load in the CSF sample could be the cause. Previous research has found that the GeneXpert MTB/RIF assay has a low NPV. Therefore, it is recommended by WHO to do further diagnostic test in GeneXpert MTB/RIF negative suspected TBM patients [30]. WHO recommended the GeneXpert MTB/RIF Ultra test for diagnosis of TB in January 2017. The Ultra assay for the detection of MTB offers improved analytical sensitivity when compared to the original GeneXpert MTB/RIF assay. The use of Ultra assay will be especially beneficial in the diagnosis of TB cases with low bacilli load (paucibacillary TB) [8].

The study's limitations included the use of conventional ZN staining rather than fluorescent staining because the latter has higher sensitivity and specificity, the use of only one drug in MGIT DST, and the inclusion of patients on anti-tubercular drugs.

## Conclusions

Our study found that the sensitivity of GeneXpert MTB/RIF assay is lower when compared to culture, so using this assay alone is not recommended. The overall performance of GeneXpert MTB/RIF assay is noteworthy. The assay has a median time to results of less than two hours compared to MGIT culture's three to four weeks. In cases where the GeneXpert MTB/RIF assay is negative, culture must still be performed. The GeneXpert MTB/RIF assay is a potentially accepted test for making an earlier diagnosis, and treatment should start right once it detects MTB.
